# Meniscal Allograft Transplantation Does Not Prevent or Delay Progression of Knee Osteoarthritis

**DOI:** 10.1371/journal.pone.0156183

**Published:** 2016-05-26

**Authors:** Catherine Van Der Straeten, Paul Byttebier, Annelies Eeckhoudt, Jan Victor

**Affiliations:** 1 Musculoskeletal Sciences and Technology, Department of Surgery and Cancer, Imperial College London, London, United Kingdom; 2 Department Orthopaedics and Traumatology, Ghent University Hospital, Ghent, Belgium; Mayo Clinic Minnesota, UNITED STATES

## Abstract

**Background:**

Meniscal tears are common knee injuries. Meniscal allograft transplantation (MAT) has been advocated to alleviate symptoms and delay osteoarthritis (OA) after meniscectomy. We investigated (1) the long-term outcome of MAT as a treatment of symptomatic meniscectomy, (2) most important factors affecting survivorship and (3) OA progression.

**Methods:**

From 1989 till 2013, 329 MAT were performed in 313 patients. Clinical and radiographic results and MAT survival were evaluated retrospectively. Failure was defined as conversion to knee arthroplasty (KA) or total removal of the MAT.

**Results:**

Mean age at surgery was 33 years (15–57); 60% were males. No-to-mild cartilage damage was found in 156 cases, moderate-to-severe damage in 130. Simultaneous procedures in 118 patients included cartilage procedures, osteotomy or ACL-reconstruction. At a mean follow-up of 6.8 years (0.2–24.3years), 5 patients were deceased and 48 lost (14.6%), 186 MAT were in situ (56.5%) whilst 90 (27.4%) had been removed, including 63 converted to a KA (19.2%). Cumulative allograft survivorship was 15.1% (95% CI:13.9–16.3) at 24.0 years. In patients <35 years at surgery, survival was significantly better (24.1%) compared to ≥35 years (8.0%) (p = 0.017). In knees with no-to-mild cartilage damage more allografts survived (43.0%) compared to moderate-to-severe damage (6.6%) (p = 0.003). Simultaneous osteotomy significantly deteriorated survival (0% at 24.0 years) (p = 0.010). 61% of patients underwent at least one additional surgery (1–11) for clinical symptoms after MAT. Consecutive radiographs showed significant OA progression at a mean of 3.8 years (p<0.0001). Incremental Kellgren-Lawrence grade was +1,1 grade per 1000 days (2,7yrs).

**Conclusions:**

MAT did not delay or prevent tibiofemoral OA progression. 19.2% were converted to a knee prosthesis at a mean of 10.3 years. Patients younger than 35 with no-to-mild cartilage damage may benefit from MAT for relief of symptoms (survivorship 51.9% at 20.2 years), but patients and healthcare payers and providers should be aware of the high number of surgical re-interventions.

## Introduction

Meniscal tears are common injuries. The overall incidence is unknown but the surgical incidence is reported to be 60 to 70 per 100.000 per year [1–3]. Most meniscal tears are caused by the combination of tissue degeneration and high mechanical loads. Meniscal function may also be lost because of traumatic events, with or without ligament injury, or congenital malformation. Meniscectomy through arthrotomy of the knee used to be a common orthopaedic treatment for symptomatic meniscal tears [4]. The advent of arthroscopy significantly reduced the morbidity of the operative procedure [5,6]. However, the appraised virtues of this new technology including its technical elegance, decreased postoperative pain, improved aesthetics and faster recovery, lowered the threshold to enter the knee joint and perform diagnostic and therapeutic interventions, such as ‘partial’ meniscectomy. Consequently, the number of procedures increased exponentially [7] and nowadays patients often undergo repetitive knee arthroscopies with progressive loss of meniscal volume. In the US, the 2006 National Survey of Ambulatory Surgery reported approximately one million knee arthroscopies yearly including nearly 500,000 procedures for meniscal tears mostly partial meniscectomies [8].

After meniscectomy, the tibiofemoral contact area is decreased leading to higher contact stresses between the curved femoral condyle and the flat tibial plateau often associated with symptoms including pain and instability and a faster progression of tibiofemoral osteoarthritis (OA) [9]. The first report on the relation between meniscectomy and cartilage degeneration with bone remodeling dates back to 1948 [10]. In later years, the meniscus was recognized as an essential structure for biomechanical and biological homeostasis of the knee [11,12]. In a loaded, ex-vivo setting, the meniscus reduces the stress on the cartilage by load sharing of up to 50% on the medial and 70% on the lateral side [13]. The medial meniscus is an important contributor to knee stability [14] whilst both menisci play a role in shock absorption [15] and joint lubrication [16]. Recent research revealed an essential biological role of the meniscus in the development and progression of OA. In vitro pro-inflammatory stimulation of injured meniscal tissue causes production of cytokines, chemokines, matrix degrading enzymes and other catabolic factors by meniscal cells [17–19]. This effect may be enhanced by obesity and/or age-related dysregulation of anabolic gene expression and changes in cytokine release [17,20,21]. In vivo, Petty et al demonstrated radiographic signs of OA at 8 to 16 years post-meniscectomy [22] whilst Cohen et al showed cartilage loss on MRI, 7 years after meniscectomy [23].

Meniscal substitution by meniscal allograft transplantation (MAT) has been advocated to alleviate clinical symptoms and delay the development and progression of OA especially in young people [24–26]. MAT follow-up studies have shown encouraging short-term results regarding healing of the allograft to the joint capsule [24] and symptomatic relief [25] and suggested protection of the articular cartilage [26]. However, none of them could demonstrate that MAT re-established the load distribution function of the native meniscus and was able to defer degenerative OA [27,28].

At Ghent University Hospital, MAT has been performed since 1989 as a surgical option post-meniscectomy. We conducted a retrospective study evaluating the long-term clinical results, allograft survivorship and radiographic OA progression. Additional knee interventions and determinants for success/failure were assessed. Long-term effect of meniscus substitution on the development and progression of degenerative OA was investigated. Hence the research questions were: (1) What is the long-term outcome of MAT as a treatment of symptomatic meniscectomy, (2) which are the most important factors affecting survivorship and (3) does meniscal substitution delay the development and progression of OA and the need for prosthetic reconstructive surgery?

## Patients and Methods

Between 1989 and 2013, 329 MAT were performed at Ghent University Hospital by four supervising orthopaedic surgeons. Indications were young to middle-aged patients (<60 years) with moderate to severe knee symptoms (pain, swelling, instability) shortly after total meniscectomy or after a failed meniscus replacement with an artificial polyurethane meniscus (8 cases) or a collagen meniscal implant (3 cases). MAT was usually performed during or shortly after complete debridement of the meniscus (total meniscectomy) in patients with recurrent symptoms after one or several partial meniscectomies. Intact donor meniscal allografts were provided by the University Hospital Tissue Bank and were matched for size, right/left knee and lateral/medial meniscus. Donor menisci were used as fresh and viable within two weeks of procurement and storage at 37°C or had been freshly frozen at -80°C. None of the allografts were irradiated. MAT were initially performed through a mini-open surgical approach [29] but since 2007 also arthroscopically [30].

We conducted a retrospective review of all MAT. Patients consulting at the department of orthopaedics and traumatology are asked to sign a generic informed consent giving permission to use their medical data for retrospective research purposes (approved by Ghent University Hospital Ethics Committee–B670201317873).

All available demographic, surgical, clinical and radiographic data were collected retrospectively from the University Hospital Electronic Patient Dossiers (EPD). Electronic patient data recording was started in 1997. Older data from paper records were scanned and stored in the EPD and could easily be consulted. Demographic data included gender, age at surgery, weight, length, BMI and smoking history. Data related to MAT included allograft preservation method, surgical approach, lateral/medial MAT, intraoperative cartilage assessment using the Modified Outerbridge scale [31] and recording of simultaneous surgeries such as cartilage procedures (microfractures or osteochondral autograft transfer system (OATS)), high tibial osteotomy (HTO) or anterior cruciate ligament (ACL) reconstruction. Clinical data included postoperative complications, knee symptoms and subsequent treatments. Additional operations at the index knee performed at the University Hospital were registered. Only the surgical interventions related to knee morbidity were analyzed. Second-look arthroscopies for suture removal or allograft biopsies for DNA testing [32] were not taken into account, because we wanted to focus on re-interventions for clinical symptoms related to the MAT. All available pre-transplantation and consecutive postoperative radiographs were rated according to the Kellgren-Lawrence radiographic OA scale [33] by two investigators (PB, AE) independently and their evaluations compared for consistency with the radiologist’s report at the time. Patients who had not been seen at the clinic recently were contacted by phone, and were asked whether they agreed to answer a few questions on their MAT. This additional oral consent was noted in the study spreadsheet. Questions included whether the allograft was still in situ, removed and/or replaced, and if they had undergone additional knee surgeries at other hospitals. Patients with MAT in situ were asked to rate their current pain on a VAS scale from 0 to 10 (0 = no pain; 10 = extreme pain) and to describe any other knee symptoms. Besides, patient satisfaction was recorded on a VAS scale from 0 to 10 (0 = not satisfied at all; 10 = very satisfied). Patients were considered ‘lost to follow-up’ if they were not traceable even after very intensive search. Failure of MAT was defined as total removal of the allograft either by total allograft meniscectomy or during conversion to a total knee arthroplasty (TKA) or a unicompartmental knee arthroplasty (UKA) [34]. Partial allograft meniscectomy, tears (sutured or not), degeneration of the donor meniscus visible on MRI or arthroscopically, meniscal extrusion, and symptomatic knees needing additional treatment were not considered a failure as long as the allograft was not totally removed.

Statistical analyses were performed using the IBM-SPSS Statistics 22 software (SPSS, an IBM Company, Chicago, IL, USA) and SAS/STAT Statistical Analysis Software (SAS Institute Inc., Cary, NC, USA). Statistical tests were verified by a professional statistician (GB). Level of statistical significance used was 0.05.

The research questions were examined as follows: (1) Life tables and Kaplan-Meier survivorship analysis was performed for the total cohort. For the allografts in situ, outcome scores including pain and satisfaction rating by the patients, meniscal re-interventions and other subsequent knee surgeries were analysed with descriptive statistics. Comparison between different outcome groups, age groups, cartilage damage and radiographic OA grading groups regarding allograft time in situ was performed using parametric and non-parametric statistics as appropriate. (2) In order to establish determinant patient and procedure factors sub-analysis was performed by gender, age at surgery, knee compartment cartilage damage, patient BMI and smoking history, allograft preservation method, open versus arthroscopic surgery, lateral versus medial meniscal transplantation and concomitant operative procedures. Comparison of survival curves of subgroups was performed using log rank (Mantel-Cox) test. Cox proportional-hazards regression was used to establish most important determining factors for survival. (3) The evolution of OA progression was evaluated by comparing Kellgren-Lawrence OA grade on consecutive X-rays in knees with MAT in situ. The difference of radiographic OA grading between consecutive X-rays was assessed with non-parametric statistics and plotted using a linear regression model (Influence Statistics) [35]. Difference in grading was also plotted against time and estimated time to increase at least one Kellgren-Lawrence grade was computed.

## Results

329 MAT were implanted in 313 patients from 1989 till 2013. In 6 cases the MAT was replaced by a new allograft after failure, 5 patients received a MAT in both knees and 5 patients a lateral and medial MAT at the same knee (Table 1). Mean age at surgery was 33.3 years (15–57 years). Sixty percent of patients were male. Cartilage damage was assessed during the operation as no to mild cartilage damage (Outerbridge grade< III) in 156 cases (47.4%) and moderate to severe damage (grade ≥III) in 130 cases (39.5%); not noted intraoperatively in 43 cases (13.1%). There was a significant correlation between age and cartilage damage (r = 0.280; p<0.0001). Simultaneous concomitant surgeries in 118 cases (35.8%) included cartilage procedures in 52 knees (15.8%) (50 microfractures; 2 OATS), HTO in 39 cases (11.9%) and ACL reconstruction in 27 knees (8.2%). Postoperative complications occurred in 18 cases (5.5%): 6 septic arthritis, 1 severe synovitis, possibly associated with allograft rejection, 1 deep venous thrombosis, 2 cases of algoneurodystrophy, 3 arthrofibrosis needing a mobilisation under general anaesthesia and 4 complications related to HTO (3 non-unions and 1 osteomyelitis).

**Table 1:** Patient Demographics: 329 Meniscal Allograft Transplantations in 313 patients.

Gender	Male: 187 (60%)	Female: 126 (40%)
**Age at surgery**	Mean: 33,3 years (15–57 years)	
**BMI**	Mean: 24.9 (16.9–35.7)	9.1% obese (BMI ≥30.0)
**Knee**	Right: 180 (55%)	Left: 149 (45%)
**Meniscus**	Lateral: 210 (64%)	Medial: 119 (36%)
**Allograft preservation**	Viable: 137 (41.6%)	Freshly frozen: 168 (51.1%)
**Surgical approach**	Open: 258 (78%)	Arthroscopic: 71 (22%)
**Cartilage: modified Outerbridge grading**	Grade < III: 156 (47.4%)	Grade ≥III: 130 (39.5%)
**Concomitant surgeries** Simultaneous: 118 (35.8%)	Cartilage (microfractures or OATS): 52 (15.8%); High Tibial Osteotomy: 39 (11.9%); fACL reconstruction: 27 (8.2%)	

**Fig 1:**
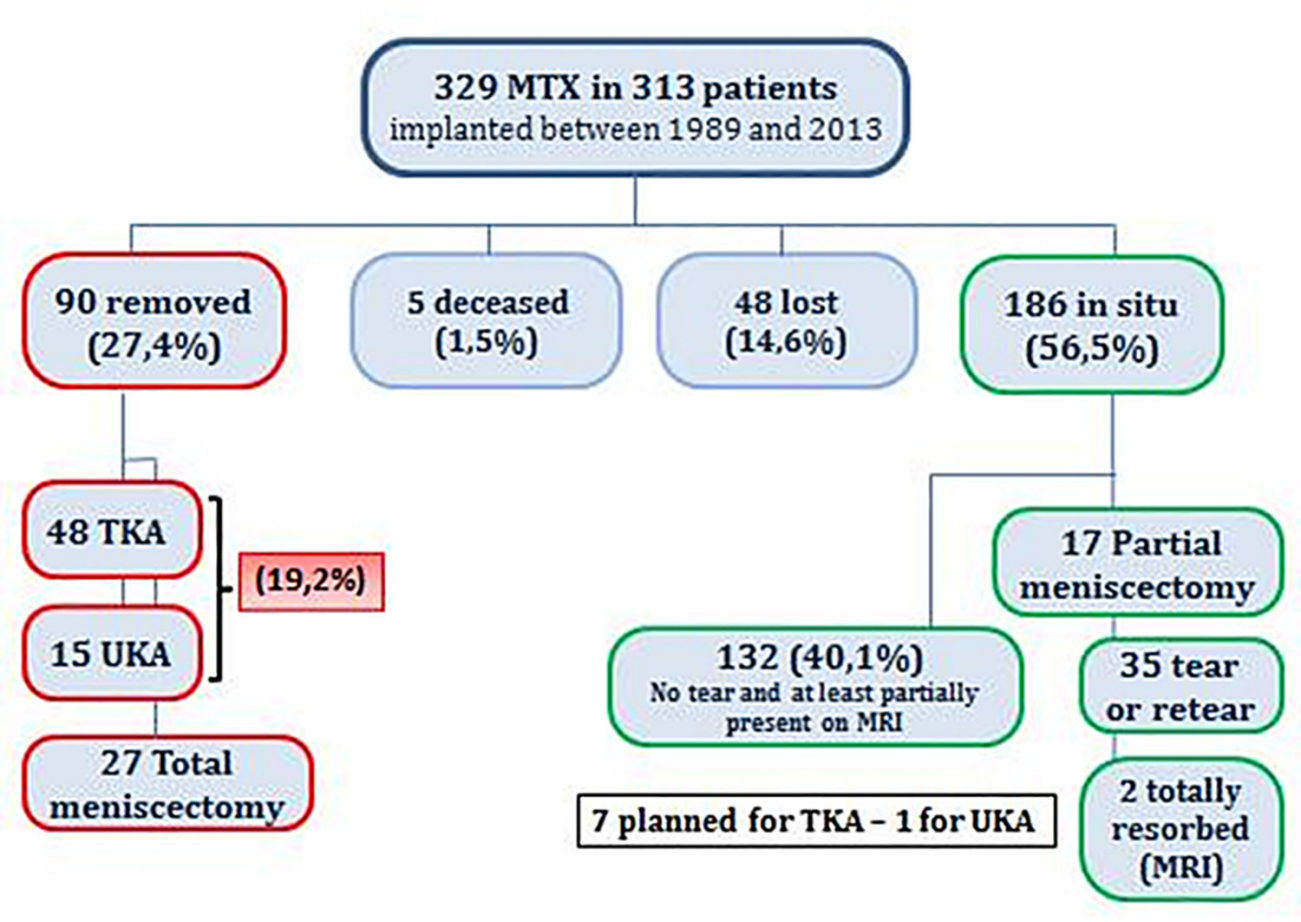
Outcomes of meniscal allograft transplantations (MAT).

(1) At the time of the retrospective review (locked-down on April 1^st^ 2015), the mean follow-up was 6.8 years (median 5.2 years–range: 2 months to 24.3 years). The outcome of MAT at last follow-up is summarized in Fig 1. Five patients died of unrelated causes and 48 (14.6%) were lost to follow-up. Ninety meniscal allografts (27.4%) had been removed or replaced after a mean of 8,5 years in situ (median 6,6 years–range 2 months to 24,0 years). Reasons for failure included pain, synovitis, knee dysfunction, meniscus tear or re-tear. There were 63 conversions to a knee prosthesis (19.2%) for OA progression: 48 TKA and 15 UKA. At the closure of the investigation 3 of these TKA had already been revised, including one for infection and 3 UKA had been converted to a TKA. In 8 additional patients with the allograft in situ a knee prosthesis was planned in the course of 2015 (7 TKA and 1 UKA) which will increase the total number of conversions to knee arthroplasty to 71 (21.6%). In 27 cases (8.2%) a total meniscectomy was performed for clinical symptoms with or without extrusion of the graft on MRI, followed by a re-transplantation with a new MAT in 6 cases.

Time till conversion to a TKA was significantly longer (mean 11.5 years; range 0.5–24.0 years; SD 6.55) compared to a UKA (mean 6.5 years; range 0.7–13.8 years; SD 4.47) (p = 0.002) or a total allograft meniscectomy (mean 3.7 years; range 0.2–13.8 years; SD 3.86) (p<0.001). Mean age at conversion to a TKA was 48.7 years (21–68 years; SD 9.17), and at conversion to a UKA 43.7 years (34–56 years; SD 6.39).

102 patients with an intact MAT in situ (33%) (no tear, resorption or partial meniscectomy), reported a median VAS for pain of 5 (mean 4.3; SD 2.7) and a median VAS for satisfaction of 8 (mean 6.7; SD 3.7) at 6.6 years mean follow-up (1.0–24.0 years). Sixteen mentioned recurrent locking, popping, instability, swelling and/or stiffness of the knee.

**Fig 2:**
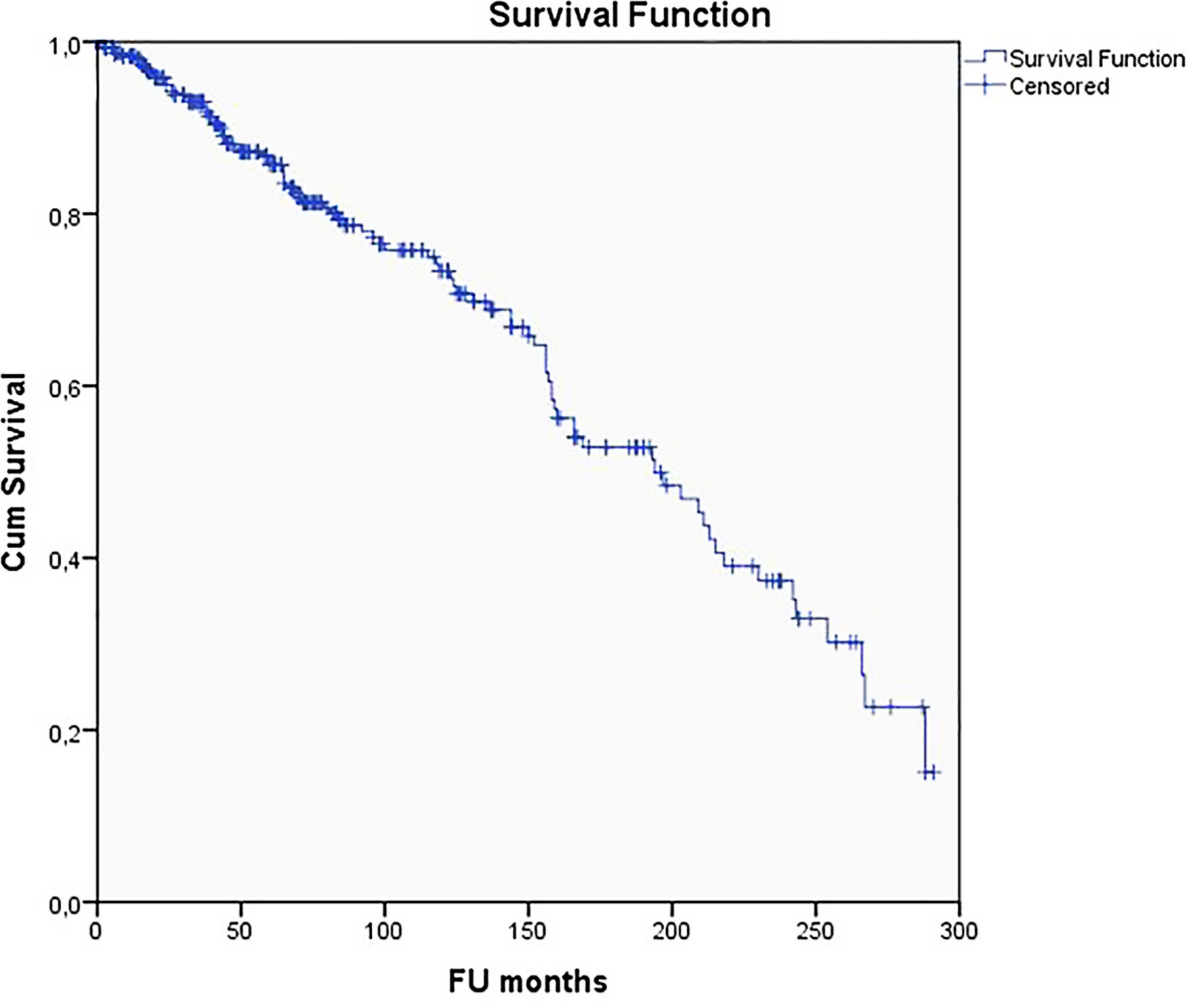
Kaplan-Meier cumulative survivorship of all MAT: endpoint removal of the allograft by total meniscectomy or during conversion to a knee arthroplasty (TKA or UKA).

Cumulative MAT survivorship with endpoint removal of the allograft (total meniscectomy or conversion to a knee arthroplasty) was 15.1% (95%CI: 13.9–16.3%) at 24.0 years (Fig 2). Mean survival time was 15.2 years.

**Fig 3:**
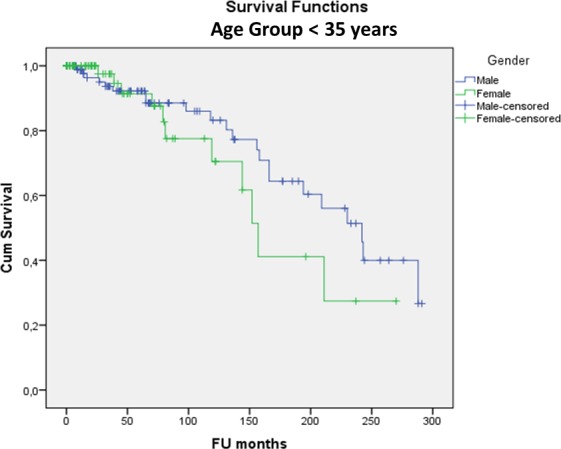
Kaplan-Meier cumulative survivorship of MAT in the <35 years at surgery group—gender: no statistically significant difference between males (blue line) and females (green line) (p = 0.262).

**Fig 4:**
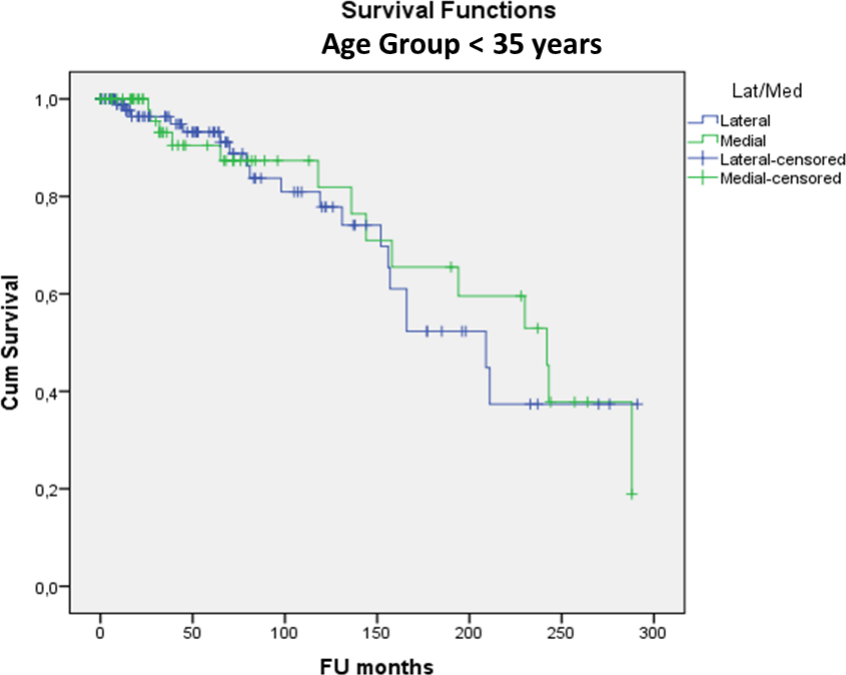
Kaplan-Meier cumulative survivorship of MAT in the <35 years at surgery group–lateral/medial: no statistically significant difference between lateral (blue line) and medial (green line) MAT (p = 0.738).

(2) There was no significant difference in MAT survivorship between genders (p = 0.552), right or left knee (p = 0.080), lateral or medial MAT (p = 0.837), BMI categories (p = 0.211), smokers or non-smokers (p = 0.235) and MAT preservation method (viable or fresh-frozen) (p = 0.118). Open versus arthroscopic surgical approach had no significant influence on survival so far (p = 0.851) but the longest follow-up time was only 7.4 years for the arthroscopic approach compared to 24.3 years for the open approach (p<0.001). In patients younger than 35 at surgery, survival was significantly better (24.1% (95%CI: 21.4–26.7%)) at 24 years; mean survival time 16.7 years) compared to older patients (8.1% (95%CI: 7.2–9.0%)) at 24 years; mean survival time 13.8 years) (p = 0.017). The odds ratio of MAT failure was 2.3 in the ≥35 years group compared to the <35 group. Yet, even in the group <35 years at surgery, 12% had been converted to a KA (15TKA/6UKA) at a mean of 13 years post-MAT for TKA and 9 years post-MAT for UKA. In the group <35 years at surgery there was no difference in survivorship between genders (at 24 years Males: 26.6% (95%CI: 23.3–29.9%); Females: 27.4% (95%CI: 21.2–33.6%)—p = 0.262) (Fig 3) or lateral versus medial meniscus (at 24 years Lateral: 37.4% (95%CI: 31.5–43.3%) Medial: 18.9% (95%CI: 15.8–22.0%)—p = 0.738) (Fig 4).

**Fig 5:**
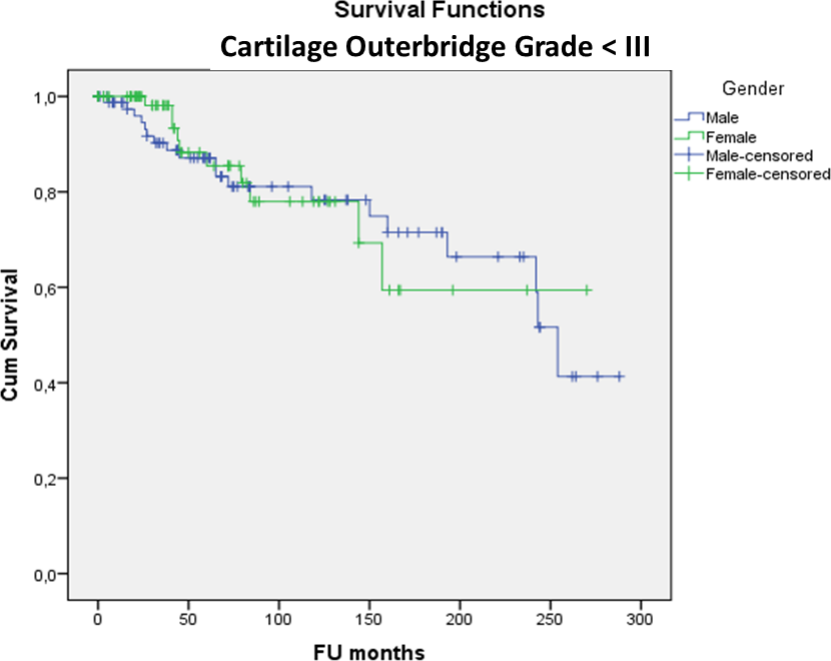
Kaplan-Meier cumulative survivorship of MAT in the cartilage Outerbridge grade < III group—gender: no statistically significant difference between males (blue line) and females (green line) (p = 0.901).

**Fig 6:**
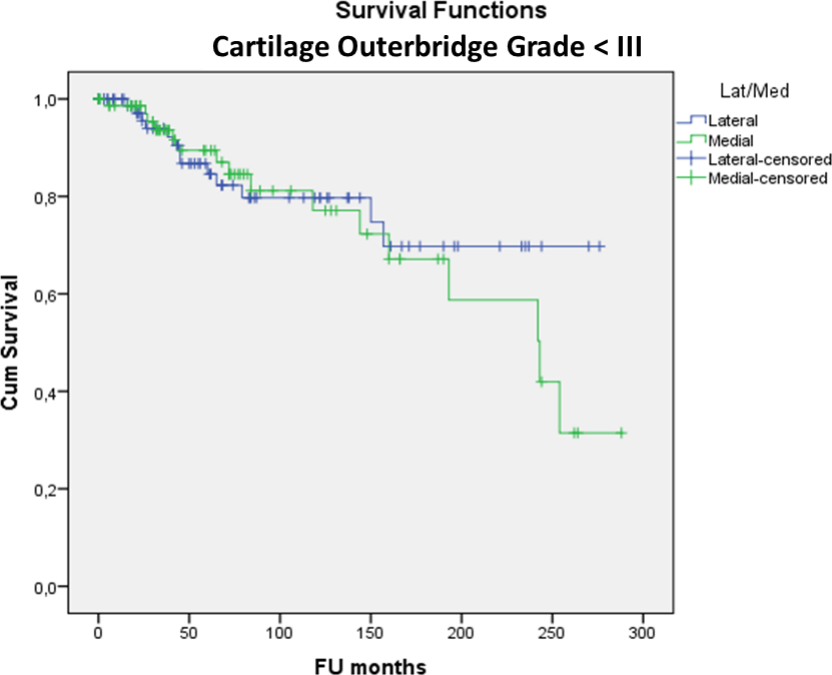
Kaplan-Meier cumulative survivorship of MAT in the cartilage Outerbridge grade < III group–lateral/medial: no statistically significant difference between lateral (blue line) and medial (green line) MAT (p = 0.591).

In knees with no-to-mild cartilage damage (Outerbridge grade <III) more allografts survived (43.0% (95%CI: 38.4–47.6%) at 24 years; mean survival time 17.6 years) compared to moderate-to-severe damage (Outerbridge grade ≥III) (6.6% (95%CI: 5.8–7.4%) at 24 years; mean survival time 13.4 years) (p = 0.003). The odds ratio of MAT failure was 3.7 in the grade ≥III group compared to <III. In the group with no to mild cartilage damage at surgery there was no difference in survivorship between genders (Males: 41.3% (95%CI: 36.0–46.6%) Females: 59.4% (95%CI: 48.5–70.3%) at 24 years—p = 0.901) (Fig 5) or lateral versus medial meniscus (Lateral: 69.8% (95%CI: 60.7–78.9%) Medial: 31.5% (95%CI: 26.7–36.3%) at 24 years—p = 0.591) (Fig 6).

Simultaneous HTO significantly deteriorated survival (0% at 24.0 years, mean survival time 11.2 years, range: 2 months to 24 years) (p = 0.010). Concomitant microfractures or ACL reconstruction did not have a significant influence on MAT survivorship (p = 0.983 and p = 0.272 respectively). In the group with concomitant microfractures 11 out of 50 were converted to a TKA (22%).

**Fig 7:**
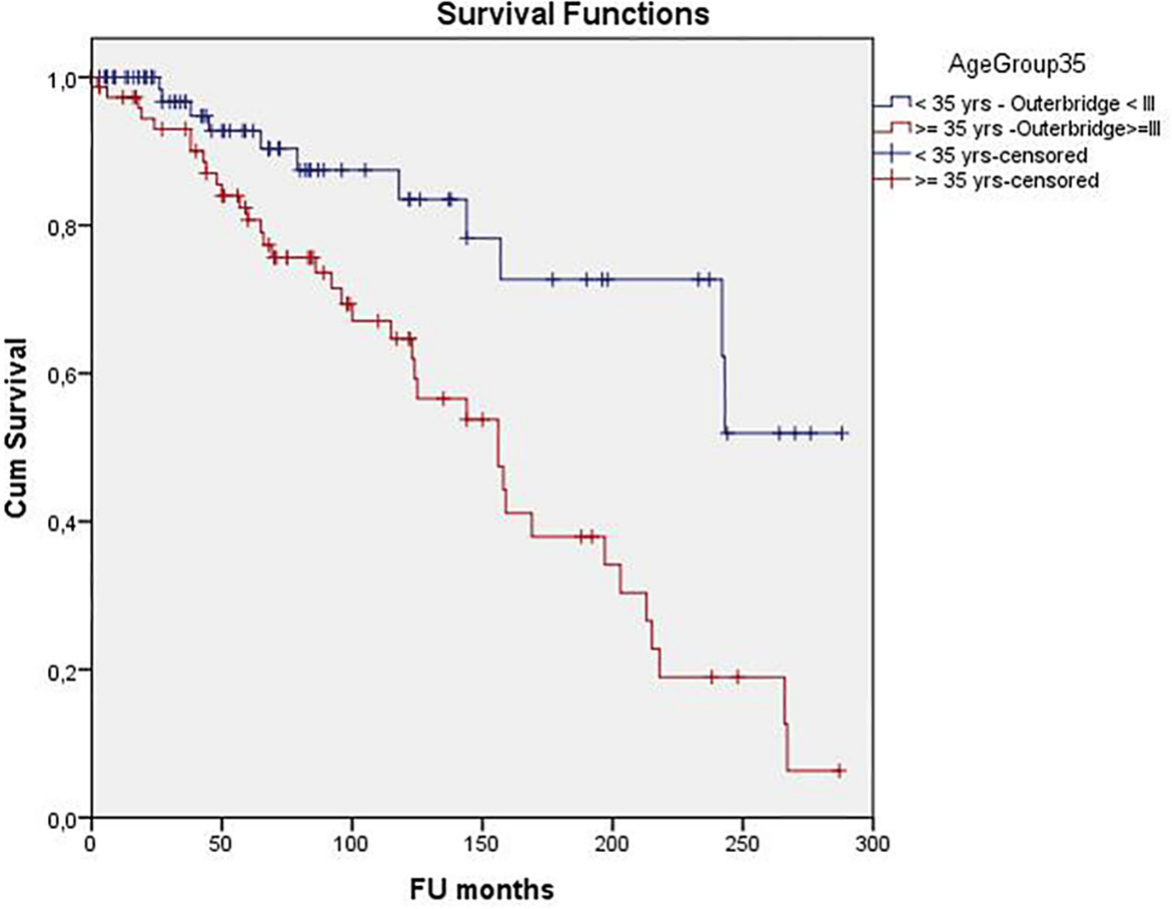
Kaplan-Meier survivorship curve (blue line) of the best case scenario (age <35 years at surgery and cartilage Outerbridge grade < III: n = 85) versus Kaplan-Meier survivorship curve (red line) of the worst case scenario (Age ≥35 years at surgery and Cartilage Outerbridge grade ≥ III: n = 77).

61% of patients underwent one or more surgical re-interventions (30% two or more, 14% three or more subsequent operations; mean 2.0; range 1 to 11) at the University Hospital for clinical symptoms at the index knee after MAT. Some patients underwent multiple further knee surgeries at other hospitals. Cox regression analysis indicated that age ≥35 years at surgery and cartilage grade ≥III were the most important determinants of failure of MAT. Best case scenario were patients <35 years at surgery with no to little cartilage damage (Outerbridge grade <III) (n = 85) with a cumulative survivorship of 51.9% (95%CI 45.0–58.8%) at 20.2 years (mean survival 19 years) whilst worst case scenario were patients ≥35 at surgery with moderate to severe cartilage damage (grade III-V) (n = 77) with a survival of 6.3% (95%CI 5.3–7.3%) at 22.3 years (mean survival time 12.7 years) (Fig 7).

**Fig 8:**
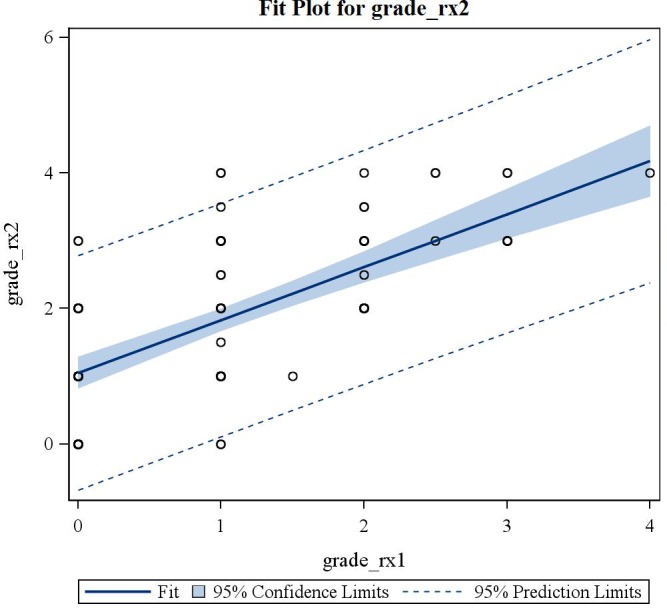
Fit-plot regression curve for evolution of Kellgren-Lawrence grade on consecutive radiographs.

**Fig 9:**
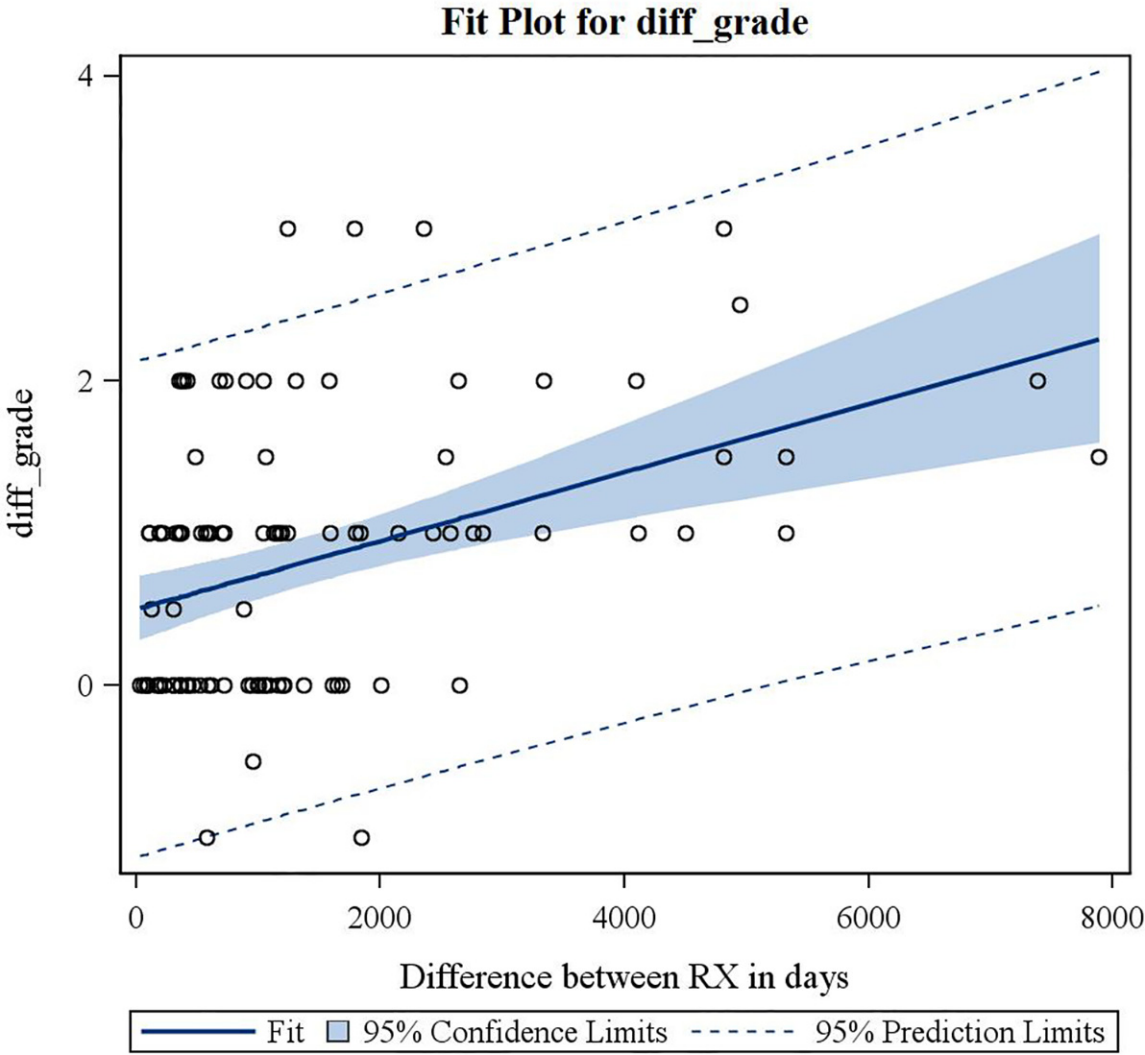
Fit-plot regression curve for difference in Kellgren-Lawrence grade on consecutive radiographs over time.

**Fig 10:**
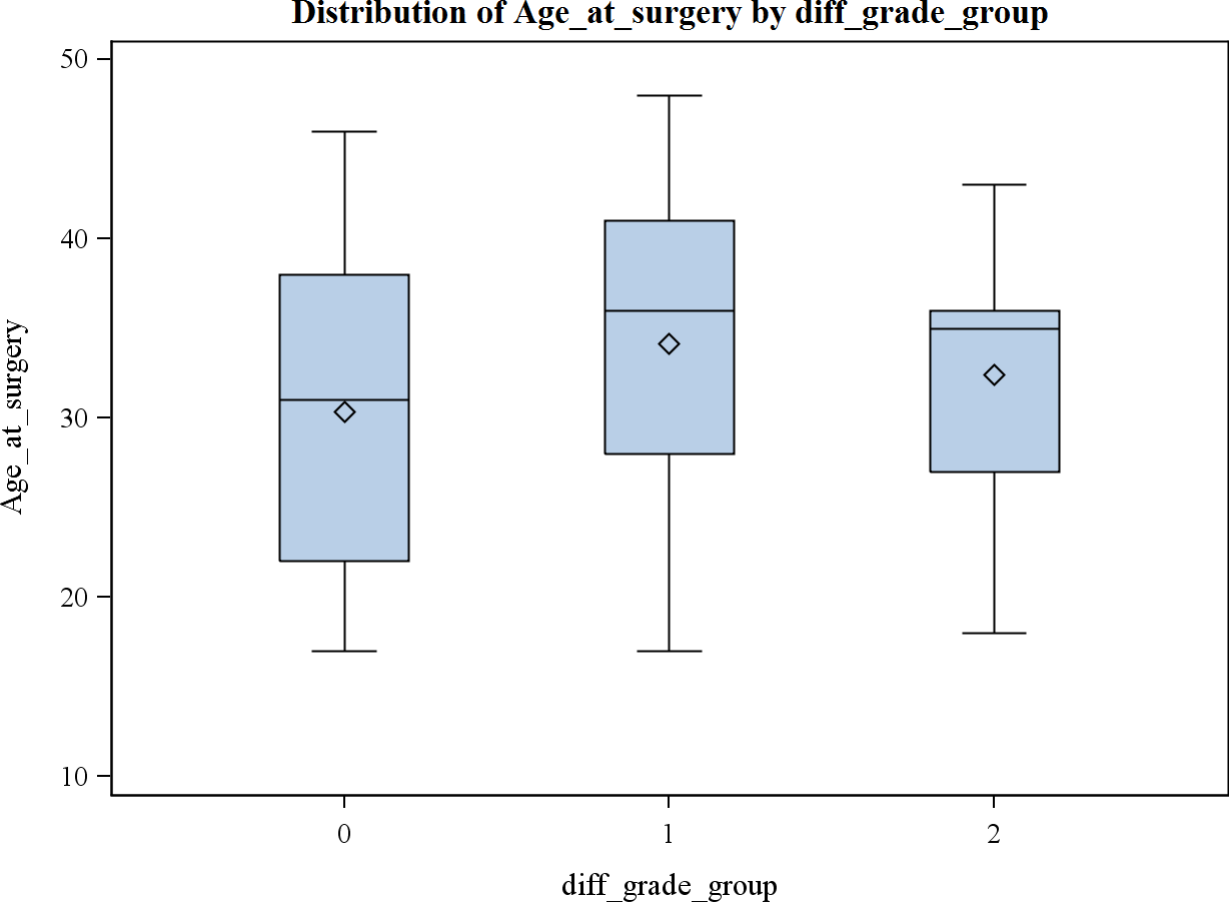
Distribution of age versus progression of grade of radiographic OA on the Kellgren-Lawrence scale (+ 0, 1, or 2 grades): no statistically significant difference (p = 0.109).

(3) Consecutive standard radiographs of 107 knees with MAT in situ showed significant tibiofemoral OA progression on the Kellgren-Lawrence scale with time (p<0.0001) (mean 1385 days (3.8 years); range 1 month to 21.6 years; SD 4.2 years—no evolution in 40.2%; grade +1 in 34.6%; grade +2 in 20.6%; grade +3 in 4.7%) (Fig 8). The mean incremental gradient was +1.1 Kellgren-Lawrence grade per 1000 days (2,7yrs) (Fig 9). In cases with concomitant microfractures, there was a trend towards a slower evolution of OA with an increased Kellgren-Lawrence grade of +0.7 per 1000 days. The group of patients who evolved more than 2 grades on the Kellgren-Lawrence scale had a higher BMI distribution (p = 0.02). There was no significant difference in progression of OA on the Kellgren-Lawrence scale with age (p = 0.109) (Fig 10), gender (p = 0.951) or lateral versus medial MAT (p = 0.539).

## Discussion

Meniscus substitution with a MAT has been advocated to alleviate symptoms of pain and instability after (partial) meniscectomy and to delay OA progression [24–26]. Pengas et al prospectively followed a cohort of 53 adolescents who had undergone meniscectomy [36]. Additional surgery was not performed till OA progression necessitated arthroplasty. At a mean follow-up of 40 years, 13.2% had received a TKA. The relative risk of OA assessed radiographically with the Kellgren-Lawrence scale was 4.5 compared to the non-operated knee at 40 years [36]. Partial meniscectomy was also shown to increase the risk for development of OA although less than total meniscectomy [37]. The current retrospective study of MAT was undertaken to evaluate the outcome of meniscal replacement after meniscectomy and to assess whether progression of knee OA was effectively delayed or prevented. The main strength of our study is that it comprises the largest patient cohort treated with MAT with the longest follow-up.

The study has several limitations. Due to its retrospective nature and the long-term follow-up up to 24 years, 14.6% of patients were lost and prospective clinical scores were not available. However, despite the complexity of the retrospective evaluation, it is important to establish a comprehensive record of MAT as a treatment involving human donor transplant material. A second limitation consists in the absence of a control group receiving conservative treatment [38–40]. Several authors have demonstrated comparable relief of clinical symptoms with physical therapy versus partial meniscectomy [38–40]. Concurrently, our results demonstrate that meniscal substitution constitutes only a temporary solution for relief of symptoms after meniscectomy. In our study, MAT was usually performed during or shortly after complete debridement of the meniscus (total meniscectomy) in patients with recurrent symptoms after one or several partial meniscectomies. As the initial partial meniscectomies were often performed at other hospitals, we have no accurate data on the time between the first partial meniscectomy and the MAT. A third limitation is that OA progression was evaluated only by use of the Kellgren-Lawrence scale on standard radiographs. A standardized, consecutive MRI assessment of the articular cartilage was not available for most patients. A prospective MRI study would provide an earlier and more accurate short-term evaluation of OA evolution [41]. However, Zaffagnini et al showed no significant difference in 5 years progression of cartilage damage on MRI after meniscal substitution with a Collagen Meniscal Implant (CMI) compared to meniscectomy without meniscal replacement [42]. Moreover, in our study, we were able to demonstrate OA advancement on consecutive standard radiographs, indicating definite cartilage deterioration. Besides, progression of OA led to 19.1% conversions to a knee arthroplasty at a mean of 11.5 years for TKA and 6.5 years for UKA.

In answer to our research questions, we found that (1) Overall cumulative survivorship of MAT was 15.1% at 24 years with a mean survival of 15.2 years. Over 50% of patients with the MAT in situ reported knee pain and/or other symptoms but most of them were satisfied with the outcome. However, the high number of subsequent knee interventions for symptoms, tear or failure of the MAT is concerning as it was associated with morbidity, (temporary) disability and socio-economic costs. In this regard, it is worth considering that Katz et al showed no difference in relief of symptoms between partial meniscectomy and physical therapy versus physical therapy alone in patients with meniscal tear and OA [40]. Besides, Sihvonen et al demonstrated that partial meniscectomy as a treatment of degenerative meniscal tears was not superior to sham arthroscopy [43].

(2) Most important determinants of success of the MAT were age younger than 35 years at surgery and no to mild cartilage damage (Outerbridge grade < III). Concomitant HTO had a negative effect on survival. We could not prove a significant positive effect of simultaneous cartilage procedures on MAT survival although concomitant microfractures were associated with a trend to slower radiographic OA progression.

(3) Delay or prevention of OA by meniscal substitution after meniscectomy could not be demonstrated in this study. On the contrary, there was a significant radiographic evolution of OA. Overall, 63 MAT (19.2%) were converted to a knee prosthesis whilst 8 additional knee arthroplasties were planned (total 71 or 21.6%). Even in the group <35 years at surgery, 21 MAT (12%) were converted to a knee prosthesis (15TKA/6UKA) at a mean of 13 years post-MAT for TKA and 9 years for UKA. These results are comparable to the report of 13.2% TKA at a mean of 40 years after meniscectomy in adolescents who did not receive any additional surgical treatment [36]. In our study, the mean age of patients at knee arthroplasty was substantially younger (48.7 years for TKA; 43.7 years for UKA) compared to large series of TKA and UKA reporting mean ages at surgery of over 60 years [44,45]. In the group less than 35 years at MAT, who were converted to a KA, the mean age at knee arthroplasty was even younger: 43.2 years (21–57) for TKA and 40.7 years (34–44) for UKA.

Our results regarding OA progression are concordant with a meta-analysis concluding that the majority of studies could not demonstrate a protective effect of MAT on cartilage and that therefore, based on the current literature, MAT does not prevent or delay OA [46]. Another literature review of 24 studies cautioned that MAT may delay the progression of damaged cartilage but does not prevent degeneration of previously healthy cartilage [47]. Whether these findings are related to cartilage degeneration initiated by meniscal injury [19] or by repetitive surgical trauma [41], or to the inability of the MAT to reproduce the biomechanical and biological function of the native meniscus, remains to be investigated.

## Conclusions

Our study indicates that meniscal allograft transplantation (MAT) performs well in patients younger than 35 with no-to-mild cartilage damage. These patients may benefit from MAT for relief of symptoms, but patients and healthcare payers and providers should be aware of the high number of surgical re-interventions. There is no evidence that MAT prevents or delays tibiofemoral OA progression.
