# Correction: Meniscal Allograft Transplantation Does Not Prevent or Delay Progression of Knee Osteoarthritis

**DOI:** 10.1371/journal.pone.0219631

**Published:** 2019-07-09

**Authors:** Catherine Van Der Straeten, Paul Byttebier, Annelies Eeckhoudt, Jan Victor

Following publication of this work [1], questions were raised about whether the cited ethics approval covered the collection of samples and analyses of clinical data described in the article.

The Ethics Committee at Ghent University have clarified that authors received specific approval for this study. The published ethics statement was incomplete, however, and is hereby corrected to the following:

Patients consulting at the department of orthopaedics and traumatology are asked to sign a generic informed consent giving permission to use their medical data for retrospective research purposes (approved by Ghent University Hospital Ethics Committee–B670201317873) of patients who needed meniscal surgery in the past. All data were generated from the patient files and put anonymously in a dataset (used for this manuscript). Only the data from the patients with meniscal allograft transplant surgery were collected out of the general database (B670201317873) and added to the dataset (used for this manuscript).
